# Translating Basic Biology of Aging Into Treatments to Increase Human Healthspan

**DOI:** 10.1093/geroni/igaf122.1100

**Published:** 2025-12-31

**Authors:** Benjamin Miller

**Affiliations:** Oklahoma Medical Research Foundation, Oklahoma City, Oklahoma, United States

## Abstract

The focus of the Miller lab is to translate basic biology of aging into treatments to increase human healthspan. We focus on proteostatic mechanisms in mitochondria to increase stress resistance for slowing aging. These studies concentrate primarily on skeletal muscle to minimize age-associated sarcopenia. We use stable isotope approaches that are amenable to both pre-clinical models and human subjects as a primary means to translate findings from the basic biology of aging to clinical implementation. Particularly useful has been the isotope D2O that we use to measure synthetic processes of protein, DNA, and RNA to answer basic and translational questions in relevant aging models including cells, yeast, C. elegans, rodents, canines, and humans. In this session, I will discuss how I sought training to allow me to perform translational work and the skillsets needed to be able to bridge pre-clinical to clinical work. I will also discuss how our clinical work informs our more mechanistic experiments as “reverse-translation”. Finally, I will present the challenges we have faced while performing work along the translational spectrum, and strategies we have used to successfully recruit and retain subjects in studies that require tissue sampling.

